# Drill-Hole Bone Defects in Animal Models of Bone Healing: Protocol for a Systematic Review

**DOI:** 10.2196/34887

**Published:** 2022-07-18

**Authors:** Frederik Duch Bromer, Mikkel Bo Brent, Jesper Skovhus Thomsen, Annemarie Brüel

**Affiliations:** 1 Department of Biomedicine Aarhus University Aarhus C Denmark

**Keywords:** systematic review, animal models, preclinical, bone defect, drill-hole, fracture model, bone, bone healing, protocol, bone fracture, animal model, healing, bone healing, laboratory, laboratory animal

## Abstract

**Background:**

Bone fractures are common conditions of the musculoskeletal system. Several animal models of bone fractures have been established to help elucidate the complex process of bone healing. In the last decades, drill-hole bone defects have emerged as a method to study bone healing. Animal models of drill-hole defects are easy to standardize and do not require external fixation of the bone. However, current studies of drill-hole bone defects lack detailed descriptions of techniques and interstudy standardization.

**Objective:**

This systematic review aims to present a detailed description of the different methods used to induce drill-hole bone defects in long bones of laboratory animals and to provide a comprehensive overview of their methodology and potential for investigation of bone healing.

**Methods:**

A systematic search of PubMed and Embase will be performed of abstracts containing variations of the following four keywords: “long bone,” “drill-hole,” “regeneration,” and “animal model.” Abstract screening and full-text screening will be performed independently by 2 reviewers, and data will be extracted to a predesigned extraction protocol. The primary outcome of the included studies is the technique used to create the drill-hole bone defect, and secondary outcomes are any measurements or analyses of bone defect and regeneration. A narrative synthesis will be used to present the primary outcome, while information on secondary outcomes will be displayed graphically. The study protocol follows the PRISMA-P (Preferred Reporting Items for Systematic Review and Meta-analysis Protocols) guidelines.

**Results:**

Abstract and full-text screening is ongoing and is expected to be completed by October 2022. Data extraction will commence immediately after, and the manuscript is expected to be completed by December 2023. The systematic review will follow the PRISMA statement.

**Conclusions:**

The strength of this systematic review is that it provides a comprehensive methodological overview of the different drill-hole methods and their advantages and disadvantages. This will assist researchers in choosing which model to use when studying different aspects of bone healing.

**Trial Registration:**

International Prospective Register of Systematic Reviews CRD42020213076; https://tinyurl.com/bp56wdwe

**International Registered Report Identifier (IRRID):**

PRR1-10.2196/34887

## Introduction

Bone fractures are a common condition of the musculoskeletal system. A recent study reported an incidence rate of 3.6 fractures per 100 person years and a lifetime fracture prevalence of 38.2% for any fracture [1]. Most fractures heal easily if the fracture is sufficiently stabilized [2]. However, bone regeneration is a complex process involving multiple different cells and tissues [3-5]. Because of its complexity, sometimes, the healing process fails, and this may lead to fractures healing slowly or not at all [6]. Delayed fracture healing is more often seen in patients with comorbidities such as osteoporosis, diabetes, or old age [7-9]. In fact, as many as 5%-10% of the fractures devolve into delayed bone healing [10,11]—either as a significantly increased healing time or as a complete lack of healing resulting in a nonunion fracture. Patients with nonunion fractures experience lower quality of life than those with diabetes mellitus, stroke, or AIDS [12].

Owing to the complexity of fracture healing, studies performed in animals are often used to investigate the mechanisms of bone healing and test potential new treatment regimens [13,14]. There are multiple advantages of studying diseases in animal models [15]. Through a controlled environment and a homogenous population, disease pathology and temporal development can be studied more thoroughly than is possible in humans [16].

The bone structure comprises two types of bone tissue: cortical bone constitutes the compact shell surrounding the bone, while trabecular bone forms a porous network of interconnected bone found in the medullary space of metaphyseal and epiphyseal bone [17]. Healing of cortical and trabecular bones differs; cortical bone heals through both endochondral and intramembranous ossification [4], while trabecular bone heals through direct membranous bone formation [18].

Numerous methods of inducing bone fractures have been established in animal models [19], from resection of a bone segment [20,21] to fractures obtained by 3-point bending [22]. Most of these studies investigate healing at the diaphysis of a long bone [18,23]. Recently, drill-hole bone defects have been used increasingly as a model of bone injury [24-27]. While this method is less directly translational to clinical fractures, they nevertheless have several advantages in basic research: they are better suited for investigation of trabecular bone healing in, for example, the metaphysis, they are easier to standardize and require no external fixation of the bone [25]. In most fracture models, it is difficult to achieve uniform fracture fixation, and variations in fracture fixation are bound to occur. Fracture fixation is a crucial factor of optimal bone healing [5], and elimination of this variable is a major advantage of the drill-hole methods.

Currently, there is little consistency in the methodology of the drill-hole bone defects. Therefore, the purpose of this systematic review is to present a narrative synthesis of the different animal models of long bone drill-hole bone defects and their potential use in preclinical and translational research.

## Methods

### Overview

This review is registered with the PROSPERO (International Prospective Register of Systematic Reviews; CRD42020213076) and has been written in accordance with the current guidelines of the PRISMA-P (Preferred Reporting Items for Systematic review and Meta-analysis Protocols) guidelines [28].

### Eligibility Criteria

#### Overview

This systematic review aims to provide a detailed description of the different methods used to induce drill-hole bone defects in long bones of laboratory animals and to provide a comprehensive overview of their methodology and potential for investigation of bone healing. This research question has been formulated following the Population, Intervention, Comparison, and Outcome (PICO) framework [29].

#### Population

This review will include all in vivo animal studies using drill-hole bone defects.

#### Intervention

All types of drill-hole bone defects generated in long bones will be included.

#### Comparator or Control Group

Studies will be included if they comprise a control group with a drill-hole defect that receives no treatment to influence healing of the defect, or if they encompass an unoperated or a sham-operated control group.

#### Outcome

The primary outcome is the surgical procedure used to generate the drill-hole injury and the anatomical location of the defect. Secondary outcomes are healing time and methods used to analyze the healing of the bone defect.

### Information Sources and Search Strategy

A systematic literature search will be performed in the PubMed and Embase databases without date restriction. The search strategy consists of 4 blocks:

- First block: specifies that only long bones will be investigated.
- Second block: specifies that only drill-hole defects are included.
- Third block: specifies that a secondary outcome of bone regeneration must be investigated.
- Fourth block: specifies that all animal species can be included using a search filter for PubMed and Embase [30].

The search strategy (Textbox 1) aims to include all original animal studies of drill-hole defects as a disease model of bone fracture. The search string has been developed in cooperation with an expert information specialist of systematic reviews. Furthermore, free-hand searches in Google Scholar will be performed, and any relevant articles will be included.

**Search strategy for PubMed and Embase.**

**First block:**

- PubMed: (“long bone”[Tiab] OR “long bones”[Tiab] OR tibia*[Tiab] OR “Tibia”[Mesh] OR fibul*[Tiab] OR “Fibula”[Mesh] OR femur*[Tiab] OR femor*[Tiab] OR “Femur”[Mesh] OR metatar*[Tiab] OR “Metatarsal Bones”[Mesh] OR phalanx*[Tiab] OR “Finger Phalanges”[Mesh] OR “Toe Phalanges”[Mesh] OR “Humerus”[Mesh] OR humeru*[Tiab] OR humera*[Tiab] OR radius[Tiab] OR “Radius”[Mesh] OR “Radius”[Mesh] OR ulna*[Tiab] OR “Ulna”[Mesh] OR metacar*[Tiab] OR “Metacarpal Bones”[Mesh] OR diaphysis[Tiab] OR diaphyses[Tiab] OR “Diaphyses”[Mesh] OR epiphysis[Tiab] OR epiphyses[Tiab] OR “Epiphyses”[Mesh])
- Embase: (“long bone”:ti,ab,kw OR “long bones”:ti,ab,kw OR tibia*:ti,ab,kw OR 'tibia'/exp OR fibul*:ti,ab,kw OR 'fibula'/exp OR femur*:ti,ab,kw OR femor*:ti,ab,kw OR ‘femur’/exp OR metatar*:ti,ab,kw OR 'metatarsal bone'/exp OR phalanx*:ti,ab,kw OR 'phalanx'/exp OR humeru*:ti,ab,kw OR humera*:ti,ab,kw OR 'humerus'/exp OR radius:ti,ab,kw OR 'radius'/exp OR ulna*:ti,ab,kw OR 'ulna'/exp OR metacar*:ti,ab,kw OR 'metacarpal bone'/exp OR diaphysis:ti,ab,kw OR diaphyses:ti,ab,kw OR 'diaphysis'/exp OR epiphysis:ti,ab,kw OR epiphyses:ti,ab,kw OR 'epiphysis'/exp)

AND

**Second block:**

- PubMed: (drill*[Tiab] OR burr*[Tiab] OR bur[Tiab] OR circular[Tiab])
- Embase: (drill*:ti,ab,kw OR burr*:ti,ab,kw OR bur:ti,ab,kw OR circular:ti,ab,kw)

AND

**Third block:**

- PubMed: (Heal*[Tiab] OR regener*[Tiab] OR growth[Tiab] OR repa*[Tiab] OR formati*[Tiab] OR osteogenesis[MeSH] OR “bone regeneration”[MeSH])
- Embase: (Heal*:ti,ab,kw OR regener*:ti,ab,kw OR growth:ti,ab,kw OR repa*:ti,ab,kw OR formati*:ti,ab,kw OR 'bone development'/exp OR 'bone regeneration'/exp)

AND

**Fourth block:**

- PubMed: Search filter by van der Mierden et al [30]
- Embase: Search filter by van der Mierden et al [30]

### Data Management and Selection Process

#### Overview

All studies found through the search strategy will be uploaded to the web-based screening and data extraction tool *Covidence*, and titles and abstracts will be screened by 2 independent reviewers. Prior to abstract screening, the 2 reviewers will practice screening of 50 abstracts to ensure a uniform screening. Then, the included studies will undergo full-text screening for eligibility by the same reviewers. Any disagreements over eligibility will first be discussed internally by the 2 reviewers, and if no agreement can be reached, the eligibility will be decided by an independent arbitrator. Following were the screening inclusion and exclusion criteria.

#### Animals or Population

- Inclusion criteria: Animal studies where a drill-hole defect is created in a long bone (all species, sexes, and ages).
- Exclusion criteria: Nonanimal studies, human studies, and in vitro or ex vivo studies.

#### Intervention or Exposure

- Inclusion criteria: Studies creating a drill-hole defect in any long bone. All types of drills, burrs, or other instruments creating circular bone defects will be included.
- Exclusion criteria: Bone defects created without usage of a drill, burr, or similar instruments or any bone defect that needs fixation. As this is a review of drill-hole models used as a bone healing model and not as a model of osseointegration, all studies with permanent implants placed in a drill hole (titanium, screws, etc) will be excluded.

#### Comparator or Control

- Inclusion criteria: Studies including a control group. Either a healthy control group not subjected to a drill-hole defect or a control group subjected to the drill-hole defect without treatment of the defect.
- Exclusion criteria: Studies not using a control group as described above.

#### Outcome Measures

- Inclusion criteria:
  Primary outcome: Information about the anatomical location of the defect, type of defect, defect size, number of defects, and depth of the defect.
  Secondary outcomes: Information about defect repair, including healing time (only for untreated groups), bone characteristics (dual energy x-ray absorptiometry [DEXA], computed tomography [CT], µCT, histomorphometry, mechanical strength, etc).
- Exclusion criteria: No relevant information about the method of defect creation. No follow-up of the healing defect.

#### Publication Type

- Inclusion criteria: Full-text original research papers.
- Exclusion criteria: Any type of review, meta-analysis, or conference abstract.

#### List of Exclusion Criteria for Screening

- Not a full-text study
- Study not written in English
- Not an original animal study
- No defect is created
- The defect is not created by a drill- or burr-like technique
- The defect is not created in a long bone
- The bone injury is fixated
- No control group is included
- No relevant outcome is obtained as follow-up on the defect healing

### Data Collection Process

#### Overview

All eligible studies will have relevant data extracted by a reviewer to a predesigned data extraction protocol in *Covidence*. To ensure uniform data extraction and to reduce the risk of error in the data extraction process, 2 independent reviewers will perform data extraction on 10 full-text studies prior to full data extraction. The extracted data will be compared to verify that there is no disparity in the extraction. Should there be differences in the extracted data, the data extraction protocol will be refined, and the reviewers will perform full-text screening on 10 new full-text studies as a quality check.

#### Data Items and Availability

Data extracted from articles will include study characteristics (type of study, sample size calculations, duration of the study, number of groups, and number of animals per group), animal characteristics (species, strain, sex, age, genetic modifications, and body weight), method of drill-hole creation (description of drill methodology, type of drill-hole, size of the drill-hole, anatomical bone and site, number of defects, type of drill, drill speed, and depth of the drill-hole), and method of analysis (initial and final defect size, healing time [only for untreated groups], DEXA, CT, *µ*CT, histomorphometry, and mechanical strength). This list is not exhaustive and may be updated upon refinement of the extraction protocol or during full-text data extraction. Data from graphs and figures will be collected with assistance of a web-based tool. All data from the data extraction protocol will be available upon request from the corresponding author.

#### Bias Assessment

The focus of this systematic review is the available methods of drill-hole bone defects in the literature and not the treatment of bone defects. Therefore, we include the healing time of any untreated group, as we believe this is an important aspect of any bone injury model. However, since we do not compare or analyze any treatment effect, no assessment of treatment bias is planned. To ensure uniformity between groups prior to creation of the bone defect, allocation method of animals (both blinding and randomization), sample size calculation, and baseline characteristics (sex, age, weight, species, strains, housing conditions, and provider of the animals) will be assessed. No analysis of meta-bias will be conducted.

#### Data Synthesis

The main outcome of this systematic review is methods used for creating drill-hole bone defects. Therefore, a narrative synthesis will be performed to describe all methods of creating bone defects in the included studies. Data on animal models will be tabulated to show similarities and differences in technique, anatomical location, and bone healing between the included drill-hole models clearly. Furthermore, data related to the main outcome (type of model, anatomical bone or site, and animal species or strain) will be presented graphically in bar or pie charts for improved clarity. The type of drill-hole injury and drill-hole site will also be presented and subdivided by animal species. Finally, the advantages and disadvantages of the injury model, bone site, and animal selection will be presented in the Discussion section.

#### Confidence in Cumulative Evidence

This is a narrative synthesis of animal models for preclinical investigation of bone healing. As such, no assessment is planned.

## Results

Abstract and full-text screening is ongoing and is expected to be completed by October 2022. Data extraction will commence immediately after, and the manuscript is expected to be completed by December 2023. The study is expected to be published in a peer-reviewed journal once work is complete. The systematic review will follow the PRISMA statement [31].

## Discussion

### Expected Findings

Owing to the complexity of bone healing and its different healing processes, no single animal model can be used to study all aspects of the process. Therefore, the availability of different models and knowledge of their advantages and disadvantages allows researchers to choose the best-suited model based on their research question.

Drill-hole bone defects are relatively new methods used to investigate bone healing. These methods may help elucidate some of the mechanisms of bone healing in the diaphysis and especially in the metaphysis of long bones, and the interaction between cortical and trabecular bone healing. Understanding the healing processes in trabecular bone and the differences and similarities compared to the healing processes in cortical bone is highly relevant [18]. Hip fractures are common in older or osteoporotic individuals and involve healing of both cortical and trabecular bones in the proximal femoral metaphysis [32,33]. Until recently, most animal studies of fracture healing were performed at the middiaphysis, where little or no trabecular bone is found. Therefore, drill-hole defect models may increase options for preclinical studies of metaphyseal fractures, where healing of both trabecular and cortical bones can be studied. However, literature on drill-hole models suffer from a lack of standardization between studies, and often only inadequate descriptions of the applied technique are available.

Therefore, this review aims to present a systematic overview of the drill-hole methods, describe the methodologies and techniques, and highlight their potential to elucidate aspects of the bone healing process. In the future, the review will hopefully assist researchers in selecting an appropriate model when planning their study protocols.

### Strengths and Limitations

The strength of this systematic review is that it will present a comprehensive methodological overview of the different drill-hole methods and their advantages and disadvantages, and that abstract and full-text screening will be performed by 2 independent reviewers to increase reproducibility.

One limitation of this study is that some abstracts found from the search string are not written in English and cannot be included owing to the lack of linguistic proficiency in those languages in our research team. Another limitation is that owing to current lack of standardization, relevant articles may not be found if the abstract does not sufficiently describe the method of inducing a drill-hole bone defect.

## Abbreviations

CT: computed tomography
DEXA: dual energy x-ray absorptiometry
PICO: Population, Intervention, Comparison, and Outcome
PRISMA-P: Preferred Reporting Items for Systematic review and Meta-Analysis Protocols
PROSPERO: The International Prospective Register of Systematic Reviews
